# Biosemantics guided gene expression profiling of Sjögren’s syndrome: a comparative analysis with systemic lupus erythematosus and rheumatoid arthritis

**DOI:** 10.1186/s13075-017-1400-3

**Published:** 2017-08-17

**Authors:** Nirav R. Shah, Braxton D. Noll, Craig B. Stevens, Michael T. Brennan, Farah B. Mougeot, Jean-Luc C. Mougeot

**Affiliations:** Department of Oral Medicine, Carolinas Medical Center, Carolinas HealthCare System, P.O. Box 32861, Charlotte, NC 28232-2861 USA

**Keywords:** Sjögren’s syndrome, Systemic lupus erythematosus, Rheumatoid arthritis, Gene expression, Meta-analysis, Concept profile analysis

## Abstract

**Background:**

Sjögren's syndrome (SS) shares many clinical and pathological similarities with systemic lupus erythematosus (SLE) and rheumatoid arthritis (RA). These autoimmune diseases mostly affect women. In this study, concept profile analysis (CPA) and gene expression meta-analysis were used to identify genes potentially involved in SS pathogenesis.

**Methods:**

Human genes associated with SS, SLE, and RA were identified using the CPA tool, Anni 2.1. The differential mRNA expression of genes common to SS and SLE (SS-SLE) was determined in female peripheral blood mononuclear cells (PBMCs) using NCBI-GEO2R. Differentially expressed (DE) SS-SLE PBMC genes in common with the SS-SLE CPA-identified genes were analyzed for differential expression in salivary glands or synovial biopsies, and for genes common to SS and RA and SLE and RA, analyzing differential expression in salivary glands in SS, synovial fibroblasts in RA, and synovial fluid in SLE. Among common genes, DE genes found in salivary gland mRNA expression in patients with SS were used for gene enrichment and SS molecular network construction. Secondary analysis was performed to identify DE genes unique to the disease site tissues, by excluding PBMC and CPA common DE genes to complement the SS network.

**Results:**

We identified 22 DE genes in salivary gland datasets in SS that have not previously been clearly associated with SS pathogenesis. Among these, higher levels of checkpoint kinase 1 (*CHEK1*), V-Ets avian erythroblastosis virus E26 oncogene homolog 1 (*ETS1*), and lymphoid enhancer binding factor 1 (*LEF1*) were significantly correlated with higher matrix metalloproteinase 9 (MMP9) levels. Higher MMP9 levels have been implicated in degradation of salivary gland structural integrity, leading to hypo-salivation in patients with SS. Salivary gland mRNA expression of *MMP9* and the expression of cytokine *CXCL10* were higher in patients with SS. CXCL10 has been shown to increase MMP9 expression and therefore may also play an important role in SS pathogenesis.

**Conclusion:**

Using CPA and gene expression analysis, we identified factors targeting MMP9 expression and/or function, namely CHEK1, CXCL10, ETS1, LEF1, and tissue inhibitor of metalloproteinase 1; altered mRNA expression of these could increase expression/activity of MMP9 in a concerted manner, thereby potentially impacting SS pathogenesis.

**Electronic supplementary material:**

The online version of this article (doi:10.1186/s13075-017-1400-3) contains supplementary material, which is available to authorized users.

## Background

Sjögren’s syndrome (SS) is a chronic autoimmune disease affecting up to 3% of the population [1]. SS is primarily characterized by dysfunctional exocrine glands due to lymphocytic infiltration, resulting in excessive dry mouth (xerostomia) and dry eyes (xerophthalmia) [2]. Autoimmune diseases often share common clinical and pathological features such as innate immune response activation, chronic inflammation, development of specific autoantibodies, and systemic dysfunction of multiple organs [1, 3, 4]. SS is most closely associated with the two autoimmune disorders, systemic lupus erythematosus (SLE) and rheumatoid arthritis (RA) [5]. Autoimmune diseases are usually more common in women. In particular, SS and SLE overwhelmingly affect women, with a ratio of women to men of 9 to 1 [6–9]. RA also affects more women than men but less drastically (ratio of 2-3 to 1) [9, 10].

Despite overlapping pathophysiological markers shared among SS, SLE and RA, the exact mechanism responsible for the onset and progression of these diseases is not fully understood [9, 11]. In recent years, in the search for biomarkers unique to SS or common between SS, SLE, and RA, several meta-analyses studies have been performed to compare multiple SS gene expression datasets with each other or in conjunction with SLE and RA datasets [11–13]. In these studies, gene expression analyses were conducted using data from peripheral blood mononuclear cells (PBMCs) or from the biopsies of tissues affected in each disease, i.e., the salivary glands (SGs) in SS, and synovial biopsies in SLE and RA [11–13].

Most previous meta-analyses studies have focused on identification of the genes that demonstrate the largest fold change in messenger RNA (mRNA) expression among SS patient samples compared to controls. Large fold changes in transcriptional expression of certain genes observed in these studies, however, could be irrelevant to disease etiology. The large fold changes may be characteristic of disease progression during advanced stages rather than at disease onset or during pre-symptomatic stages. For example, high levels of type I interferon (IFN)-related genes (e.g., *IFN-α*) are expressed in PBMCs and SG biopsies from patients with SS [14, 15]. However, increased levels of this cytokine in the SGs could be largely attributed to lymphocytic infiltration [14, 15] and not directly related to the SS etiological mechanisms initiated in the SGs. Indeed, having the recently identified potential disease susceptibility genes [2, 16, 17], along with infection by viruses with high tropism for the exocrine glands, are conditions suspected to play important roles in the etiology of SS ahead of the development of systemic autoimmune responses [18, 19].

Moreover, SS predominantly occurs in women and an X chromosome dosage effect has been recently identified [20]. Previous meta-analysis studies comparing SS, SLE, and RA mostly used gene expression data from both male and female patients [11, 12]. There is a mounting body of evidence suggesting that higher susceptibility to SS in women could be associated with the aberrant expression of specific genes located on the X chromosome in conjunction with X-linked epigenetic events, possibly involving the activation of endogenous retroviruses [21–25].

The use of concept profile analysis (CPA) based on biosemantics text-mining has emerged as a promising approach for biomedical discoveries especially when the amount of data is limited or inadequate, limited categories of controls are used, or there is a lack of general understanding in disease mechanisms [26–28]. Similar to Gene Ontology (GO) analysis, in CPA each biological entity (e.g., genes, diseases, symptoms, pathways, chemicals, drugs, tissues, or toxins) can represent a concept of a profile belonging to another concept and be ranked in the order of relevance within a list, thus defining a hierarchy based on literature mining [26–28]. Concept profiles can be matched against the human genome using data mining tools [26].

In this study, we used CPA to establish lists of genes relevant to SS, SLE, and RA with the goal of identifying novel candidate biomarkers of SS etiology or markers critical to the development of SS. Genes common to SS, SLE, and RA, and genes unique to either disease, were identified using publically available gene expression datasets.

## Methods

### Concept profile analysis using the Anni 2.1 program

Anni 2.1, an online concept-mining tool, was used to perform CPA. This program systematically retrieves literature that contains two concepts of interest such as “gene” and “disease”, in an abstract, and ranks the genes from highest to lowest occurrence using a vector space model to generate the ranked association scores [26, 27]. A higher score is assigned to greater occurrence of a particular gene and queried disease, corresponding to a higher degree of association [26]. Scoring by Anni 2.1 also identifies pairs of concepts that are not found together in an abstract but are associated with a third concept occurring in a PubMed abstract with either concept of the pair (co-occurrence) [26].

We used Anni 2.1, first, to obtain concept profiles related to the three concepts “Sjögren’s syndrome”, “systemic lupus erythematosus”, “rheumatoid arthritis” and associated published literature. These concept profiles were matched to Homo sapiens genes (Anni 2.1 embedded human genome database) thereby producing lists of genes ranked based on their degree of association with each disease concept.

After processing of duplicates and errors in the three Anni 2.1 output listings, cutoffs in gene ranking (i.e., 250, 500, 1000, 2500, or 5000 genes) were tested to determine appropriate stringency that would limit non-specific over-representation, and at the same time, would optimize pathway-related gene enrichment procedures in downstream *in silico* functional genomics analyses. The 2500 gene cutoff retrieved for each disease (i.e*.*, SS, SLE, and RA) provided appropriate stringency. The three lists of 2500 genes were overlaid using a Venn diagram generator (http://bioinfogp.cnb.csic.es/tools/venny/index.html) [29] to determine subsets of genes common to all three diseases, genes common to pairs of diseases, and genes unique to each disease. Subsets of genes were used to determine differential expression of each gene, using publically available gene expression databases, and to investigate their SS-related biological functions by using GO and molecular network analysis programs.

The Anni 2.1 PubMed database latest update had been performed in 2010. Thus, to include additional discovered genes from 2010 to 2016 in the three gene listings, manual PubMed searches were conducted using keywords corresponding to disease concepts with the highest Anni 2.1 association scores (Additional file 1: Table S1).

### Gene expression analysis of PBMCs and primary disease sites in women

Gene expression datasets obtained from PBMCs or tissue biopsies from patients with SS, SLE, or RA and from controls were retrieved by searching the National Center for Biotechnology Information-Gene Expression Omnibus (NCBI-GEO) database using the terms “Sjögren’s syndrome”, “systemic lupus erythematosus”, and “rheumatoid arthritis” through May 2016 (Table 1) [30].

**Table 1:** Gene expression datasets used for meta-analysis of SS, SLE, and RA

Disease	GEO accession	Female patients	Female controls	Tissue type
SS	GSE48378	11	16	PBMCs
SLE	GSE10325	14	11	PBMCs (CD4^+^ T cells/CD19^+^ B cells)
RA	GSE15573	14	10	PBMCs
SS	GSE23117	10	5	Minor salivary gland (MSG)
SS	GSE40611	17	12	Parotid gland (PG)
SS	GSE40568	5	3	Labial salivary gland (LSG)
SLE	GSE36700	4	2	Synovial biopsy
RA	GSE7669	5	4	Synovial fibroblast

Gene expression datasets with their corresponding disease are listed by Gene Expression Omnibus (GEO) accession numbers. For all datasets, the total numbers of patients in the disease and control groups and their tissue types are listed. *SS* Sjögren’s syndrome, *SLE* systemic lupus erythematosus, *RA* rheumatoid arthritis, *PBMC* peripheral blood mononuclear cell

To select gene expression datasets used in our study, the following criteria had to be met: (1) the gene expression dataset had to be generated from biological samples obtained from human subject cases and controls that were age-matched overall; and (2) due to the higher incidence of SS, SLE, and RA in women, either the dataset had to contain female subjects only, or the male subjects had to be removed for further analysis.

Out of 16 gene expression datasets for SS, 21 for SLE, and 27 for RA, only one PBMC dataset for each disease met all the criteria (GSE48378 for SS, GSE10325 for SLE, GSE15573 for RA). The RefSeq gene IDs in the dataset GSE48378 were converted to gene symbols using the gene ID conversion tool (g:Profiler) [31].

For disease site-specific analyses, five among 64 gene expression datasets were retrieved from the NCBI-GEO database. Three datasets selected for SS were from minor SGs (MSG) [GEO:GSE23117] [32], labial SGs (LSG) [GEO:GSE40568] subset of minor salivary glands) [33], and parotid glands (PG) [GEO:GSE40611] [34] (Table 1). Datasets selected for SLE and RA were from synovial biopsies [GEO:GSE36700] and synovial fibroblasts [GEO:GSE7669], respectively (Table 1).

For all selected datasets, differentially expressed (DE) genes in representative tissues of women with SS, SLE, or RA were identified using the online web application GEO2R (http://www.ncbi.nlm.nih.gov/geo/geo2r/) [30]. For individual probes of candidate genes identified by Anni 2.1 and the manual PubMed searches, fold changes or average fold changes in expression were determined from the GEO2R LogFC data from one or more datasets. To ensure that the gene expression data analyses were unaffected by genes represented by multiple probe values, the same probe was used for each gene across all datasets.

### Gene enrichment and functional network analysis

DE genes were selected for enrichment if they exhibited markedly different fold changes (≥1.5 or ≤ -1.5) in at least two of the three SS SG datasets (i.e.*,* GSE23117, GSE40611, or GSE40568), and were not DE in the opposite direction in the third dataset. Selected DE genes were enriched using GO biological processes and the Kyoto Encyclopedia of Genes and Genomes (KEGG; http://www.genome.jp/kegg/) pathways functional analysis module in GeneCodis (http://genecodis.cnb.csic.es/) [35]. To identify functional associations between the SS, SLE, and RA enriched subsets, the “Search Tool for the Retrieval of Interacting Genes/Proteins-database” (STRING-db; http://string-db.org/) [36] server was utilized. To expand on our functional network and to provide complementary connections within and between gene clusters, we included the major single nucleotide polymorphism (SNP)-containing candidate genes associated with SS disease susceptibility identified in two independent genome-wide association studies (GWAS) [2, 16].

### Strategy for combined CPA and gene expression analyses

**Fig. 1:**
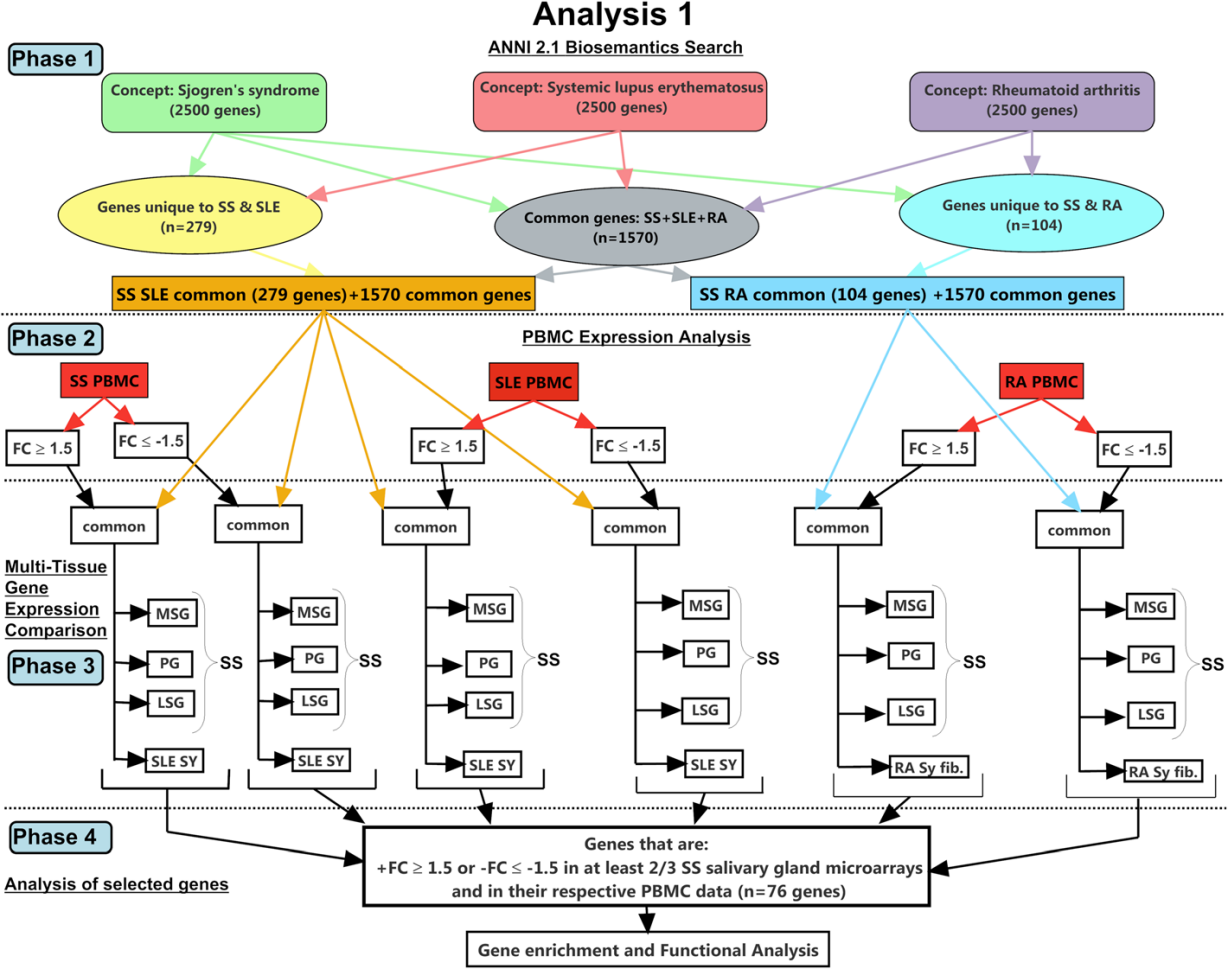
Flowchart representing the study workflow strategy. *Phase 1*: concept Profile Analysis (CPA) of human genes associated with each concept, “Sjögren’s syndrome” (*SS*), “systemic lupus erythematosus” (*SLE*), and “rheumatoid arthritis” (*RA*), and finding common genes between SS-SLE and SS-RA. *Phase 2*: comparison of common genes from phase 1 with peripheral blood mononuclear cell (*PBMC*) datasets for each disease, SS, SLE, and RA. The criteria for comparison per disease were female subjects only, and a gene expression cutoff of ≥ +1.5 or ≤ -1.5 fold changes (*FC*). *Phase 3*: comparison of gene expression of selected common genes from the PBMC and disease site (e.g., salivary gland for SS) datasets to identify differentially expressed (DE) genes. *Phase 4*: total 76 DE genes were identified from salivary gland datasets from patients with SS and used for gene enrichment and functional analysis to generate molecular interaction network 1. *MSG* minor salivary gland, *LSG* labial salivary gland, *PG* parotid gland, *SY* synovial biopsy, *SY fib.* synovial fibroblast

Two CPA and gene expression analysis methodologies were used (i.e., analysis 1 (Fig. 1); analysis 2 (Additional file 2: Figure S1)). Analysis 1 (Fig. 1) consisted of 4 phases: phase 1 included the CPA and matching to the human genome by prioritizing the comparison of SS with SLE and SS with RA; phase 2 consisted of the analysis of mRNA differential gene expression in PBMCs from female patients using NCBI GEO datasets (Table 1) to determine genes common to the three diseases and those uniquely common to SS and SLE or SS and RA; phase 3 extended the gene expression analysis to disease sites (i.e., NCBI GEO datasets obtained from SGs for SS, synovial biopsies for SLE, and synovial fibroblasts for RA) for PBMC DE genes in female patients with SS, SLE, or RA determined in phase 2; and phase 4 corresponded to gene enrichment and functional analyses using computational systems biology tools. While analysis 1 (Fig. 1) followed a directional process from CPA to gene expression analysis, analysis 2 followed a directional process from gene expression analysis to CPA (Additional file 2: Figure S1).

## Results

Our overarching goal was to identify candidate biomarkers of SS (the focus of this study) by using two approaches, namely analysis 1 (Fig. 1) and analysis 2 (Additional file 2: Figure S1), each consisting of 4 phases. In analysis 1, we derived genes associated with SS, SLE, and RA by using CPA (i.e*.*, Anni 2.1) and compared those genes to the DE genes of the SS, SLE, and RA PBMC datasets (phases 1 and 2). DE genes found to be in common were further evaluated against SS, SLE, and RA DE genes belonging to the disease sites (e.g., salivary glands for SS) (phase 3). We identified genes DE in PBMCs and at least two SS SG datasets, excluding genes differentially expressed in the opposite direction in a third SS SG dataset. Further, to prevent the possibility of missing SS DE genes in SGs but not in PBMCs, analysis 2 was performed by eliminating the PBMC DE genes and keeping only SG DE genes. We also performed a comparison between SLE and RA (complementary analysis) for information purposes, using the same approach as in analysis 1. A summary of the results is presented subsequently.

### Analysis 1 - phase 1: knowledge-based correlation analysis of genes associated with SS, SLE and RA

**Fig. 2:**
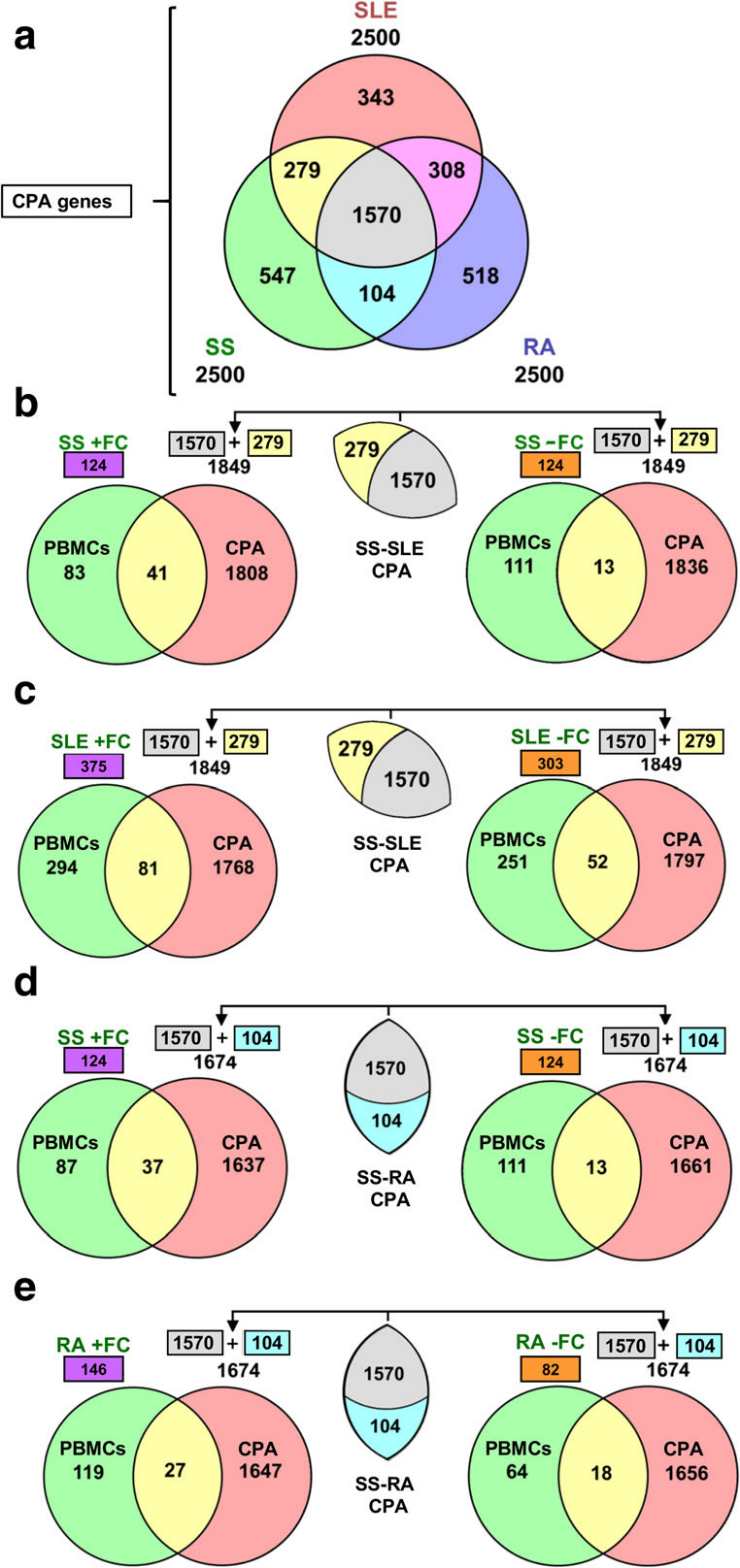
Comparison of common genes identified by matching concept profile analysis (*CPA*) against differentially expressed genes of peripheral blood mononuclear cell (*PBMC*) gene expression datasets of patients with Sjögren’s syndrome (*SS*), systemic lupus erythematosus (*SLE*), and rheumatoid arthritis (*RA*). **a** The lists of 2500 genes per disease (i.e*.*, SS, SLE, and RA), found by the text mining tool Anni 2.1, were compiled and overlaid to generate a Venn diagram. A total of 1570 genes (62.8%) were common among the three diseases, 279 genes were found in common between SS and SLE only and 104 genes were common between SS and RA only. **b**-**e** After acquiring PBMC microarrays for all three diseases from NCBI-GEO, the genes were sorted and separated based on their gene expression variation with the fold change cutoff of ≥ +1.5 (+ fold change (*FC*)) and ≤ -1.5 (− FC) for each disease (SS + FC, SS − FC, SLE + FC, SLE − FC, RA + FC and RA − FC). **b** Selected genes from PBMC datasets for SS (SS + FC and SS − FC) were compared with the common genes between SS and SLE (1570 + 279 = 1849). **c** Selected genes from PBMC datasets for SLE (SLE + FC and SLE − FC) were compared with the common genes between SS and SLE (1570 + 279 = 1849). **d** Selected genes from PBMC datasets of SS (SS + FC and SS − FC) were compared with the common genes between SS and RA (1570 + 104 = 1674). **e** Selected genes from PBMC datasets of RA (RA + FC and RA − FC) were compared with the common genes between SS and RA (1570 + 104 = 1674)

Using the Anni 2.1 online program, concept profiles were obtained for the three diseases: SS, SLE, and RA. Our query matching the three concepts, “Sjögren’s syndrome”, “systemic lupus erythematosus”, and “rheumatoid arthritis”, with the list of human genes embedded in Anni 2.1 as “Homo sapiens genes” concept, retrieved all known human genes associated with each disease to a variable extent based on abstract occurrence in PubMed. From the ranked gene output, generated by Anni 2.1, we selected the top 2500 genes providing appropriate stringency for downstream gene GO and molecular network analysis (see “Methods”). All common and unique genes for SS, SLE, and RA found by our CPA are shown in the Venn diagram (Fig. 2a).

As shown in Fig. 2a, 1570 genes (62.8%) were common to the three related autoimmune diseases [4]. A total of 279 genes were uniquely common to SS and SLE, 104 genes uniquely common to SS and RA, and 308 uniquely common to SLE and RA. These results suggest that SS and SLE share greater similarity than SS and RA in terms of common gene representation.

### Analysis 1 - phase 2: comparative gene expression analysis of PBMCs in female patients with SS, SLE, and RA

Using CPA (Anni 2.1), 1849 genes (1570 + 279) were found in common between SS and SLE and 1674 genes (1570 + 104) in common between SS and RA (Fig. 2a). Table 1 lists the NCBI-GEO PBMC gene expression datasets used in our analysis of each disease. The NCBI GEO2R online R-based expression analysis tool was used to identify DE genes (fold changes (FC) ≥ +/- 1.5 in up and down directions) in female patients compared to female controls (Venn diagram, Fig. 2b-e). Particular focus on comparing SS to SLE, identified a total of 248 DE genes in SS, 124 upregulated (UR) (Fig. 2b left), 124 downregulated (DR) (Fig. 2b right), and 678 DE genes in SLE, 375 (UR) (Fig. 2c left), and 303 (DR) (Fig. 2c right).

The SS and SLE DE genes were then compared to the 1849 SS and SLE common genes determined by CPA (Anni 2.1) (Fig. 2a) with the following results: a total of 41 (Fig. 2b left) and 81 (Fig. 2c left) (UR) genes, and 13 (Fig. 2b right) and 52 (Fig. 2c right) DR genes in PBMCs from patients with SS or SLE were in common (see gene listings in Additional file 3: Tables S2-S5).

Further, specific to the comparison of SS to RA, DE genes in PBMCs from patients with SS (Fig. 2d left, 124 up; Fig. 2d right, 124 down) and DE genes in PBMCs from patients with RA (Fig. 2e left, 146 up; Fig. 2e right, 81 down) were identified. These DE genes were then compared to the 1674 SS and RA common genes as determined by CPA (Fig. 2a) with the following result: a total of 37 (Fig. 2d left) and 27 (Fig. 2e left) UR genes, and 13 (Fig. 2d right) and 18 (Fig. 2e right) DR genes in PBMCs from patients with SS or RA were found to be in common (see listings in Additional file 4: Tables S6-S9).

### Analysis 1 - phase 3: expression analysis of phase 2 candidate genes associated with SS, SLE, and RA in disease sites among female patients

After comparative gene expression analysis of SS, SLE, and RA PBMCs, we investigated the fundamental role played by the DE genes individually and collectively, primarily in SS. The primary pathological manifestation that defines SS occurs in major and minor SGs and is characterized by periductal lymphocytic infiltration of the glands resulting in destruction of acinar cells [37, 38]. SLE and RA, on the other hand, affect various tissues and have different primary pathological manifestations such as swelling and inflammation of skeletal joints [8, 39].

All DE genes in PBMCs from SS, SLE, and RA datasets that were found in common with the CPA-identified genes (phase 2 as described previously, Fig. 2 and Additional files 3 and 4: Tables S2-S9) were used to determine their differential expression in the SG datasets.

We identified 76 genes (Additional file 5: Table S10) that were differentially expressed in at least two of the three SG microarray datasets, and not in the opposite direction in the third dataset. All CPA-derived disease site expression data are shown in Additional files 3, 4, and 6: Tables S2-S5, S6-S9, S11-S14). Of the 76 genes, TIMP1 and MMP9 have been associated with SS [40, 41]. The following 22 genes have not been described clearly before as being linked to the pathogenesis of SS: *AURKA*, *CD163*, *CD74*, *CES1*, *CHEK1*, *CLEC4C, COL4A3*, *CXCL5*, *CXCR6*, *ETS1*, *IL2RB*, *ITGB1*, *LAMP3*, *LEF1*, *MKI67*, *PTGIS*, *RAD51*, *SLC18A2*, *STAT2*, *TACR1*, *TNXB*, and *TSHR*. To understand the potential role of these DE genes in the pathogenesis of SS, we used all the 76 DE genes for functional classification and molecular network-related pathway analysis.

### Analysis 1 - phase 4: functional classification and molecular network pathway analysis of phase 3 candidate genes

For functional classification and disease association of the 76 phase 2 DE genes, we used GeneCodis (http://genecodis.cnb.csic.es) for KEGG disease pathway analysis [35, 42, 43]. The GeneCodis analysis of the GO biological process revealed 16 major functional categories of gene sets (Table 2). These functional categories, including cytokine-mediated signaling, type-1 interferon (IFN) response, and response to virus, have been previously associated with SS [6, 14].

**Table 2:** Gene enrichment of SS-related pathways using GeneCodis

Annotations-biological processes	NG	Hyp_c*
1. GO:0019221: cytokine-mediated signaling pathway	15	5.95006e-17
2. GO:0006955: immune response	14	4.76772e-11
3. GO:0060337: type I interferon-mediated signaling pathway	8	1.42292e-09
4. GO:0006954: inflammatory response	11	2.46689e-09
5. GO:0007166: cell surface receptor signaling pathway	9	2.36659e-08
6. GO:0006935: chemotaxis	8	4.83921e-08
7. GO:0032496: response to lipopolysaccharide	8	7.63604e-08
8. GO:0071260: cellular response to mechanical stimulus	5	9.01203e-06
9. GO:0008284: positive regulation of cell proliferation	9	8.76728e-06
10. GO:0019882: antigen processing and presentation	5	9.656e-06
11. GO:0002544: chronic inflammatory response	3	2.33879e-05
12. GO:0045087: innate immune response	8	2.62163e-05
13. GO:0009615: response to virus	6	4.64203e-05
14. GO:0060333: interferon-gamma-mediated signaling pathway	5	5.66952e-05
15. GO:0032355: response to estradiol stimulus	5	7.95296e-05
16. GO:0007165: signal transduction	13	7.872e-05

The 76 genes, found by concept profile analysis and differentially expressed in salivary glands of patients with Sjögren’s syndrome (SS), were grouped based on Gene Ontology (GO) [34, 65]. *NG* number of annotated genes in the input list of 76 differentially expressed genes

*Hyp_c is the corrected hypergeometric *p* value

GeneCodis KEGG pathway analysis confirmed that many of the 76 genes are involved in disease and infection, including autoimmune diseases (Table 3). The functional associations between these genes were then determined by creation of a gene interaction map (molecular network) using the STRING-db web service [36]. STRING-db formulates gene maps with connections/interactions derived from both empirical evidence (including literature sourced through text mining) and functionally predicted interactions based on characteristics such as protein structure [36].

**Table 3:** KEGG pathways of SS-related DE genes associated with diseases using GeneCodis

	Items	Disease	NG	Hyp_c*	Genes
1.	Kegg:05162	Measles	12	1.25E-14	*STAT1, STAT2, OAS2, MX1, FAS, IFNG, DDX58, IL2RB, OAS1, IFIH1, TACR1, TLR7*
2.	Kegg:05160	Hepatitis C	7	4.61E-07	*STAT1, STAT2, OAS2, IFIT1, DDX58, OAS1, LDLR*
3.	Kegg:05145	Toxoplasmosis	6	5.21E-06	*STAT1, MAP2K3, IFNG, LY96, ITGB1, LDLR*
4.	Kegg:05142	Chagas disease (American trypanosomiasis)	5	3.41E-05	*FAS, IFNG, CALR, CD247, CFLAR*
5.	Kegg:05152	Tuberculosis	3	2.06E-02	*STAT1, CD74, IFNG*
6.	Kegg:05140	Leishmaniasis	3	2.67E-03	*STAT1, IFNG, ITGB1*
7.	Kegg:05146	Amoebiasis	3	7.11E-03	*IFNG, CD1D, COL5A1*
8.	Kegg:05332	Graft-versus-host disease	3	3.43E-04	*KLRC1, FAS, IFNG*
9.	Kegg:05320	Autoimmune thyroid disease	2	1.49E-02	*FAS, TSHR*
10.	Kegg:05143	African trypanosomiasis	2	9.84E-03	*FAS, IFNG*
11.	Kegg:05412	Arrhythmogenic right ventricular cardiomyopathy	2	3.22E-02	*LEF1, ITGB1*
12.	Kegg:05130	*Escherichia coli* infection	2	2.05E-02	*LY96, ITGB1*
13.	Kegg:04940	Type I diabetes mellitus	2	1.00E-02	*FAS, IFNG*
14.	Kegg:05323	Rheumatoid arthritis	2	4.09E-02	*CXCL5, IFNG*
15.	Kegg:05212	Pancreatic cancer	2	3.07E-02	*STAT1, RAD51*

List of diseases associated with the 76 differentially expressed (DE) genes in salivary glands of patients with Sjögren’s syndrome (SS) from the Kyoto Encyclopedia of Genes and Genomes (KEGG) pathway analysis. *NG* Number of genes out of the 76 DE genes

*HYP_c is the corrected hypergeometric *p* value

**Fig. 3:**
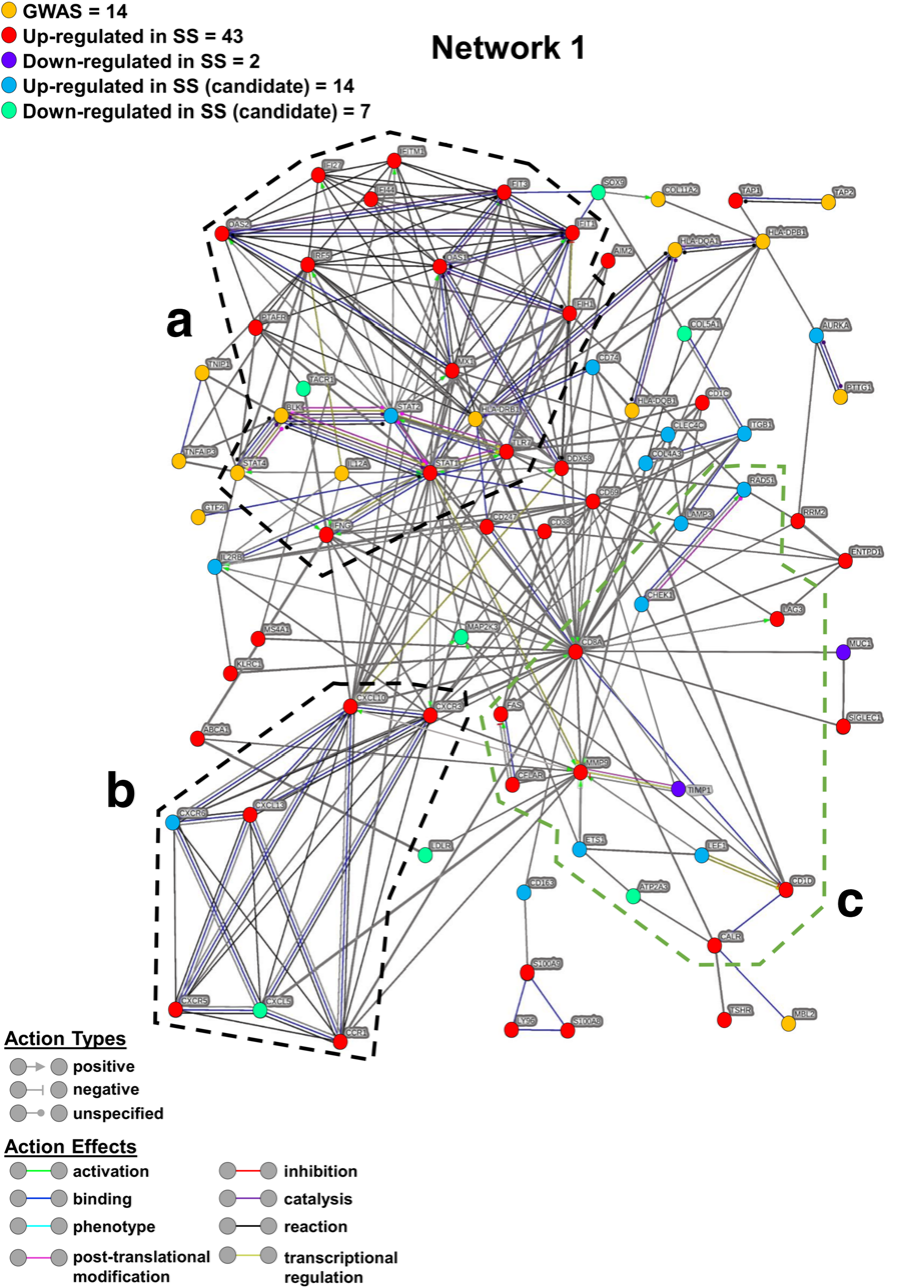
Pathway analysis of 76 selected differentially expressed genes with emphasis on three subnetworks. Using the online program STRING-db, we generated a broad interaction network from our selected 76 genes. Within the network, we assigned particular *colors* to each node (gene) to represent the expression of that particular gene in patients with SS. Node colors are explained in the figure. The *yellow nodes* are originally derived from previous genome-wide association studies (GWAS) of patients with Sjögren’s syndrome (*SS*) and are added for the validation and strengthening of our independently derived network. The *red* and *purple nodes* represent genes that are upregulated and downregulated in SS, respectively. Similarly, the *light blue* and *green nodes* represent genes exhibiting upregulation and downregulation in SS, respectively. These genes have not yet been strongly linked to SS pathophysiology. The *lines* connecting the *nodes* (*edges*) represent interactions between two nodes and can be derived from evidence or inferred from previously known data: *magenta* experimentally derived; *blue* predicted interaction through gene co-occurrence; *gray* predicted functional interaction derived from either homologous protein interactions in other species/associations in curated databases/co-mentioned in PubMed abstracts; *yellow* transcriptional regulation (experimentally derived); *black* reaction (experimentally derived); and *purple* catalysis (experimentally derived). *Edges* ending in a *green arrow*, *red bar*, or *black circle* represent an action between the two nodes that can range from positive, negative, or unspecified, respectively. Subnetworks: **A** genes of the interferon gene family and interferon-stimulated genes are outlined by a *black dashed line*; **B** genes associated with chemotaxis are outlined by a *black dashed line*; **C** genes identified in our study as potential biomarkers of SS are outlined by a *light green dashed line*

To further substantiate the relevance of our methodology and findings, we incorporated into our molecular network major genes that had previously been attributed to SS pathogenesis based on multiple GWAS [2, 16, 17]. These genes include: *TNIP1*, *TNFAIP3*, *GTF2I*, *STAT4*, *BLK*, *IL12A*, *HLA-DRB1*, *HLA-DQB1*, *PTTG1*, *HLA-DPB1*, *HLA-DQA1*, *COL11A2*, and *TAP*2. By doing so, we identified several key interactions that overlapped seamlessly with our original molecular network model (Fig. 3, network 1), giving further support to our approach. We identified both UR (n = 43) and DR genes (n = 2). Genes (n = 14) found by independent GWAS of SS are also represented in network 1 [2, 16]. Further, we found 22 DE genes (14 UR genes and 8 DR genes) that were associated with both IFN-α and chemotaxis pathways, but not yet shown to be clearly associated with SS (Additional file 5: Table S10). Of these DE genes, *CHEK1*, *ETS1*, and *LEF1* are known to increase the expression of MMP9 [44–47] which has been implicated in the pathogenesis of SS [40, 41]. In addition, *CXCL10* (Fig. 3), shown to increase the expression of MMP9 in a colorectal cancer model [47], is also part of network 1 (Fig. 3).

We further investigated the differential expression of other known regulators of MMP9 expression such as *TIMP1*, an inhibitor of MMP9 function [40, 41]. Although, *TIMP1* was not found to be DE in any of the PBMC datasets, its expression was DR 1.5-fold on average in the three SG datasets. We also determined whether any of the 76 genes identified in phase 3 were included in our network (Fig. 3) by using our set criteria for differential expression and by excluding genes with known clear association with SS (i.e*.*, the 22 genes).

Also, despite the fact that carboxylestrase 1 (*CES1*) gene was significantly DR in all three SG datasets (4.24-fold, within the top 2% on average of < - 1.5 FC DR genes in all SG datasets; Additional file 5: Table S10) and UR in PBMCs (1.63 fold; Additional file 3: Table S2), the STRING-db network analysis did not identify *CES1* (excluded from network 1; Fig. 3). This was due to the lack of connections between *CES1* and other genes part of network 1 (Fig. 3), even when using the lowest confidence level for stringency in STRING-db.

Our analysis also revealed two major biological signaling pathways (Fig. 3). First, the IFN-α pathway/immune response pathway can be attributed primarily to the significant upregulation of IFN-stimulated genes (ISGs) (subnetwork A; Fig. 3). The second major pathway, the chemotaxis initiation pathway (subnetwork B; Fig. 3), is the result of chemokine-related gene stimulation that initiates dendritic cell recruitment to SG areas. Part of the remaining genes were used to create a subnetwork related to the MMP9 regulatory pathway (subnetwork C; Fig. 3).

### Analysis 2: genes differentially expressed in SGs but not in PBMCs of patients with SS

Here, we sought to confirm some of the genes of network 1 (Fig. 3) found to be UR in analysis 1 (Additional file 2: Figure S1). First, the genes UR in at least two salivary gland datasets, and not DR in the third (n = 2769 genes) were compared with the UR genes from SS PBMC dataset (n = 303 genes) (Additional file 7: Figures S2, S3). The 118 DE genes in common were eliminated, leaving only the SG DE genes (n = 2651) (Additional file 7: Figures S2, S3). The 2651 remaining SG-specific DE genes were then compared to the 1570 CPA-identified genes common to SS, SLE, and RA, which resulted in 381 common genes (Additional file 7: Figure S4). A second network, network 2 (Additional file 8: Figure S5), was created using these 381 genes, to be compared with network 1 (Additional file 9: Figure S6). These two networks (including subnetworks A, B, and C) partially overlapped and did not significantly alter the previously stated conclusions.

Nevertheless, analysis 2 revealed certain DE genes that had not been detected in the initial analysis. Of particular interest were the genes related to the MMP9 regulatory pathway, i.e.*, SMAD3*, *TIMP2*, *TIMP3*, and *TLR4*. DE genes belonging to a subset of both networks were combined to create a composite network (Additional file 10: Figure S7), incorporating 15 genes from network 1 (subnetwork C; Additional file 9: Figure S6) and 35 genes from network 2 (subnetwork C; Additional file 8: Figure S5). This composite network includes four common genes in the MMP9 regulatory pathway, namely *CHEK1*, *ETS1*, *LEF1* and *RAD51*. Additionally, by repeating the same process and looking at DR genes from SG datasets and PBMCs, we identified 640 genes generating a much larger and complex network (n = 308 genes at the highest STRING-db confidence level) (data not shown).

### Complementary analysis: CPA of SLE and RA for determination of common genes differentially expressed in PBMCs and the respective disease site specimens

SLE datasets were compared to the RA dataets by using the same discovery process as in analysis 1 (i.e.*,* phase 1, CPA SS *vs.* SLE *vs.* RA; phase 2, CPA vs*.* PBMCs; phase 3, PBMCs vs*.* disease site; phase 4, network analysis). This analysis resulted in identification of 1878 genes (1570 + 308) in common between RA and SLE using CPA. Further, 107 genes were found to be UR and 77 genes DR based on the PBMC datasets. Comparing these 184 DE genes to the 1878 CPA-identified genes resulted in 91 common DE genes in the SLE synovial fluid dataset (67 UR and 24 DR) and 14 DE genes in the RA synovial fibroblast dataset (9 UR and 5 DR) (Additional file 6: Tables S11-S14). Further functional analysis is required to determine the relevance of these genes to the pathogenesis of both SLE and RA.

## Discussion

The pathophysiology of autoimmune disorders such as SS, SLE, and RA is complex, yet all share some common clinical features such as active innate immune response, T cell signaling, and chronic inflammation [3, 4]. The etiology of these diseases remains poorly understood [4, 23, 24], although there is a growing body of evidence that X chromosome dosage, viral infection, and retro-element activation might play an important role in the onset of SS and SLE [20, 21, 23–25].

The majority of previous approaches have focused primarily on inter-disease gene expression between SS, SLE, and RA at the expense of intra-disease gene expression. Additionally, in previously performed meta-analysis studies of SS, SLE, and RA, gene expression profiles of PBMCs only were reported [11, 12]. Other meta-analysis studies have focused on a single disease (e.g., SS, SLE, or RA) using samples from the disease site (e.g., SGs for SS, synovial fluid for SLE, or synovial fibroblasts for RA) [13, 48, 49]. A major caveat of these meta-analysis approaches is the lack of gene expression comparison between PBMCs and the primary site of disease pathology. Thus, the mechanistic changes in PBMCs, which could potentially correlate to changes at the primary site of disease or provide clues as to how these changes might govern tissue-specific autoimmunity, remain largely unexplored. Also, small expression changes in gene subsets acting in concert to significantly impact a biological disease pathway may have not been fully characterized in these previous studies [50].

To our knowledge, this is the first study combining concept mining analysis (CPA using Anni 2.1) and gene expression analysis data on PBMCs along with primary disease site tissues in female patients with SS, SLE, or RA. This study focused on SS by comparing independent mRNA expression datasets generated from PBMCs along with disease-site specimens for SS (SGs), SLE (synovial fluid), and RA (synovial fibroblasts).

Our CPA-identified genes were investigated for their differential expression in PBMC datasets across the three diseases (SS, SLE, and RA) and then further analyzed for their differential expression in datasets generated from disease-site specimens. Minute curation of datasets and the use of CPA identified 22 DE genes in female patients with SS that have not yet been clearly attributed to SS pathophysiology. Among these 22 genes, 21 (excluding *CES1*) along with the other previously identified genes involved in SS pathogenesis (e.g., *MMP9* and *TIMP1*) formed a tight molecular network (network 1) with a high level of confidence. This result is suggestive of the potential importance of these genes in SS development and progression. Our results are overall consistent with the previous findings showing differential regulation of most genes involved in various biological pathways such as IFN-α signaling, chemotaxis, or response to viral infection.

Additionally, *CES1* which was not part of network 1, was found DR by more than fourfold in SGs and UR (~1.6-fold) in PBMCs, indicating that this downregulation may not be directly associated with lymphocytic infiltration. The role of the *CES1* gene in SS pathogenesis warrants further investigation. CES1 has been linked to the pathogenesis of non-Hodgkin’s lymphoma, possibly involving a mechanism by which the downregulation or deficiency in CES1 reduces the ability of macrophages to kill cancer cells [51]. In addition, patients with SS are 44 times more susceptible to developing non-Hodgkin’s lymphoma compared to the normal population [52].

Perez et al. showed that high *MMP9*/*TIMP1* mRNA and protein ratios correlated closely with destruction of the basal lamina of acinar and periductal cells in patients with SS [40, 53]. *MMP9* came under our scrutiny because its expression is considerably higher in all of the SG-related microarray gene expression datasets, whereas its expression remained unchanged in the PBMCs of patients with SS. Our meta-analysis confirmed that *MMP9* mRNA expression is UR in the SGs of patients with SS, as has been previously described [40, 53].

We identified genes (i.e*.*, *CHEK1, ETS1*, *LEF1*, *TIMP1,* and *CXCL10*) in network 1 and/or network 2, which were DE in all SG datasets of patients with SS. These genes regulate the expression or function of MMP9 [44–46, 54–56]. *CHEK1*, *CXCL10*, *ETS1*, and *LEF1* have been previously shown to UR MMP9 expression in experimental systems in vitro [44–47, 55, 57]. Additionally, DR of *TIMP1* in all SG datasets is consistent with a previous study that showed *TIMP1* downregulation at the mRNA level, using RT-PCR, and at the protein level, using immunohistochemical assays [40]. TIMP1 downregulation at the protein level was shown to increase MMP9 function in a post-translational process [40]. Also, *RAD51* (Additional files 8, 9 and 10: Figures S5, S6, S7) is involved in DNA repair processes such as telomerase repair by direct interaction with *CHEK1*, a process that might impact X chromosome inactivation [58–61]. Furthermore, the upregulation of *ETS1* found in our study of three SG datasets, is consistent with the study by Liang et al. [58]. In this study, higher *ETS1* expression in SS was shown based on the same parotid gland dataset used in our study. The expression of *ETS1* remained unchanged in the SS PBMC dataset analyzed in our study, suggesting a role in the disease pathophysiology and/or the etiology of SS in regard to a possible pre-existing susceptibility within the SG.

In the present study, we also confirmed *CXCL10* to be one of the most significantly UR cytokines in all SS patient datasets, as compared to other cytokines such as *IL-6*, *IL-8* and *CCL5*. CXCL10 is known to increase MMP9 expression [47, 57]. Importantly, we also found *CXCL10* expression to be significantly higher in PBMCs from patients with SS (2.17 FC). In contrast *CHEK1*, *ETS1*, and *LEF1* were only found UR in the SG datasets. Notably, *TLR7*, a gene found by the complementary analysis that yielded 381 genes, was significantly UR (3.34 FC) in all SG datasets, but not in the PBMCs from patients with SS. TLR7 has been shown to upregulate expression of cytokines such as CXCL10 in SLE via recognition of nucleic acids including retro-element *Alu* RNA or foreign RNA/DNA from incoming viruses. In particular CXCL10 is known to induce MMP9 expression, which could damage the extracellular matrix (ECM) and SG cells, potentially affecting saliva secretion [62]. Further, in SGs CXCL10 is known to trigger recruitment and chemotaxis of monocytes [47]. We also analyzed the expression of *TIMP1*, a major inhibitor of MMP9 in all SG datasets of patients with SS. As anticipated, the expression of this gene was lower in the SG datasets of these patients compared to controls.

Overall, the results from both analyses raise the possibility that combined effects of candidate genes, *CHEK1, ETS1*, *LEF1*, *TIMP1,* and *CXCL10*, might lead to increased MMP9 levels that can potentially be detrimental to the structural integrity of the SGs in patients with SS. Furthermore, *TIMP1* is an X-linked gene that has been investigated for the effects of its polymorphisms in X chromosome inactivation [63, 64]. Whether *TIMP1* has any role in the imbalance of the female/male ratio remains to be determined. Because mRNA expression levels do not necessarily correlate with protein levels, biological experiments are warranted to demonstrate whether direct or combined regulatory effects of *CHEK1*, *ETS1*, *LEF1*, and *TIMP1* on MMP9 expression or function indeed occur in patients with SS.

**Fig. 4:**
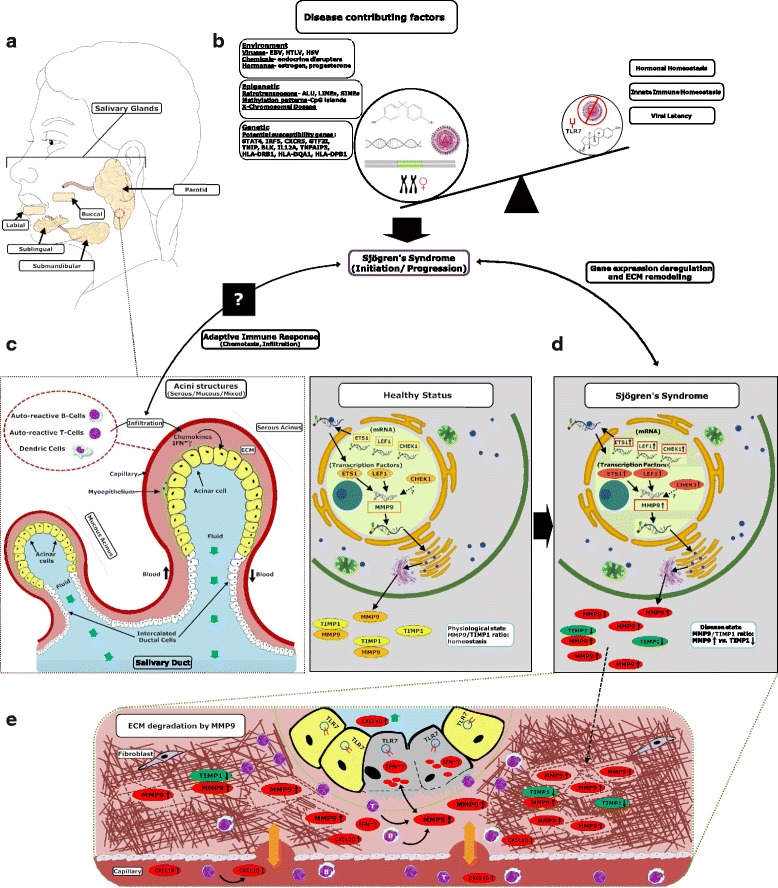
Proposed model explaining the loss of human salivary gland structural integrity in Sjögren’s syndrome (SS) based on the computational biosemantics analysis, gene expression and network analyses. **a** Major and minor salivary glands of the oral cavity. **b** Potential combinatorial factors may lead to SS. The current model involves multiple factors that together may play a role in the development of the disease. The primary factors include genetics, environmental factors, viruses, and retro-elements that may disrupt homeostasis. **c** Salivary unit portion showing individual acini. Acinar cells from these glands secrete water, salts and/or protein (major components of saliva) into the oral cavity. In SS, capillaries surrounding salivary tissue mediate the immune response by passing various interferons and chemokines produced by acinar cells into the bloodstream, which initiate the dendritic cell movement to the area. **d** Transcription factors ETS1 and LEF1 could directly upregulate (UR) matrix metalloproteinase 9 (*MMP9*) expression. MMP9, also known as gelatinase B, is involved in extracellular matrix degradation. **e** TLR7 is suggested to be a major player in the secretion of cytokines such as CXCL10 in systemic lupus erythematosus and other autoimmune diseases. MMP9 and CXCL10 feedback potentiate extracellular matrix (*ECM*) destruction. CXCL10, a cytokine, stimulates dendritic cell recruitment to a specific area while at the same time has been shown to increase MMP9 expression in a positive feedback-like mechanism. TIMP1, also known as tissue inhibitor of metalloproteinases, binds directly to metalloproteinases inhibiting their enzymatic activity. While MMP9 and TIMP1 are regulated in a ratio-specific manner, patients with SS have markedly UR levels of MMP9 and downregulated TIMP1, which might play a role in the progression of glandular destruction brought on by the disease

Based on our findings and current literature, we propose a model providing an explanation for the potential impact on the etiology and pathophysiology of SS of the candidate genes discussed (Fig. 4). In this model, we have also taken into consideration the potential role of viruses, retro-elements and other environmental factors in SS. The present study uncovered the potential impact of PBMC DE genes on tissue-specific gene expression profiles related to SS. This fundamental comparison could provide a deeper understanding of the etiology of SS or similar diseases. We have shown that combining CPA with curated gene expression datasets can be useful in identifying candidate biomarkers of complex diseases or in the targeted drug discovery process [26–28, 65].

A limitation of our analysis is the lack of uniformly normalized data across platforms and optimal controls in the original experiments. One of the eight datasets used was based on an Illumina platform and the rest were from the Affymetrix platform, indicating that the FC in expression is approximate. Also, there are fundamental differences between the cellularity and gene expression profiles of different cell types within the SG tissue, including periductal, acinar and infiltrated mononuclear cells. The microarray datasets available in GEO2R for SS have been generated from the studies that did not account for these differences in cellularity, e.g., by using a laser capture microdissection technique. Nevertheless, to find the most relevant candidate genes involved in the pathogenesis of SS, our approach compared datasets from the related autoimmune diseases, SS, SLE, and RA.

## Conclusions

Overall, our meta-analysis combining both CPA and gene expression analysis supports the hypothesis that increased levels of MMP9 resulting from dysregulation of CHEK1, ETS1, LEF1, TIMP1 and CXCL10 might contribute to the pathogenesis of SS. Further molecular and biochemical experiments are required to confirm potential biomarkers associated with the MMP9 regulatory pathway in order to better understand the etiology of this complex disease. In conclusion, targeting multiple MMP9 effectors might be a useful strategy for therapeutic development in SS.

## Additional files

Additional file 1: Table S1. Concepts profiles of SS, SLE, and RA obtained from Anni 2.1. (DOCX 17 kb)

Additional file 2: Figure S1. Flowchart explaining analysis 2. (PDF 424 kb)

Additional file :3 Tables S2-S5. Differential expression of SS and SLE CPA-identified common genes. (DOCX 79 kb)

Additional file 4: Tables S6-S9. Differential expression of SS and RA CPA-identified common genes. (DOCX 53 kb)

Additional file 5: Tables S10. Differential expression of CPA-identified genes in salivary glands of patients with SS. (DOCX 40 kb)

Additional file 6: Tables S11-S14. Differential expression of SLE and RA CPA-identified common genes. (DOCX 58 kb)

Additional file 7: Figure S2-S4. Comparison of differentially expressed genes unique to salivary glands of SS female patients with CPA identified common genes from SS, SLE, and RA. (PDF 1003 kb)

Additional file 8: Figure S5. Pathway analysis of genes common in the CPA analysis and upregulated in at least two out of three SS salivary gland datasets while not upregulated within PBMCs. (PDF 6363 kb)

Additional file 9: Figure S6. Pathway analysis of 76 selected differentially expressed genes with emphasis on three subnetworks. (PDF 9341 kb)

Additional file 10: Figure S7. Composite network of subnetwork C from network 1 (**Figure S4**) and network 2 (**Figure S6**). (PDF 6025 kb)

## Abbreviations

BLK: B lymphoid tyrosine kinase
CCL5: C-C motif chemokine ligand 5
CD19: Cluster of differentiation 19
CD4: Cluster of differentiation 4
CES1: Carboxylesterase 1
CHEK1: Checkpoint kinase 1
COL11A2: Collagen type XI alpha 2
CPA: Concept profile analysis
CXCL10: C-X-C motif chemokine ligand 10
DE: Differentially expressed
DR: Downregulated
ECM: Extracellular matrix
ETS1: V-Ets avian erythroblastosis virus E26 oncogene homolog 1
FC: Fold change
GO: Gene Ontology
GTF2I: General transcription factor IIi
GWAS: Genome-wide association study
HLA-DPB1: Major histocompatibility complex, class II, DP beta 1
HLA-DQA1: Major histocompatibility complex, class II, DQ alpha 1
HLA-DQB1: Major histocompatibility complex, class II, DQ beta 1
HLA-DRB1: Major histocompatibility complex, class II, DR beta 1
HUGO: Human Genome Organization
IFN: Interferon
IL12A: Interleukin 12A
IL-6: Interleukin 6
IL-8: Interleukin 8
ISG: Interferon stimulated gene
KEGG: Kyoto Encyclopedia of Genes and Genomes
LEF1: Lymphoid enhancer binding factor 1
LSG: Labial salivary gland
MMP9: Matrix metalloproteinase 9
mRNA: Messenger ribonucleic acid
MSG: Minor salivary gland
NCBI-GEO: National Center for Biotechnology Information-gene expression omnibus
PBMC: Peripheral blood mononuclear cells
PG: Parotid gland
PTTG1: Pituitary tumor-transforming 1
RA: Rheumatoid arthritis
SG: Salivary gland
SLE: Systemic lupus erythematosus
SNP: Single nucleotide polymorphisms
SS: Sjögren’s syndrome
STAT4: Signal transducer and activator of transcription 4
STRING: Ssearch tool for the retrieval of interacting genes/proteins
TAP2: Transporter 2, ATP-binding cassette, sub-family B
TIMP1: Tissue inhibitor of metalloproteinase
TNFAIP3: Tumor necrosis factor, alpha-induced protein 3
TNIP1: TNFAIP3 Interacting protein 1
UR: Upregulated
