# Supplementary material for: Biosemantics guided gene expression profiling of Sjögren’s syndrome: a comparative analysis with systemic lupus erythematosus and rheumatoid arthritis
Source: Arthritis Res Ther. 2017 Aug 17;19:192. doi: 10.1186/s13075-017-1400-3 (PMC5561593; doi:10.1186/s13075-017-1400-3)
Supplement: Supplementary file 1 — Concepts profiles of SS, SLE, and RA obtained from Anni 2.1. (DOCX 17 kb) [file 13075_2017_1400_MOESM1_ESM.docx]

**Table S1.** Concepts profiles of SS, SLE, and RA ranked by their relative association to each disease by Anni 2.1 program.

| **Process** | **SS** | **SLE** | **RA** |
| --- | --- | --- | --- |
| 1. B-cell activation | 5 | 7 | 29 |
| 1. Antibody formation | 7 | 5 | 14 |
| 1. Germinal center formation | 8 | 86 | 175 |
| 1. Lymphocyte activation | 11 | 13 | 17 |
| 1. Major histocompatibility complex location | 12 | 18 | 16 |
| 1. Immunoglobulin class switching | 13 | 10 | 52 |
| 1. Humoral immune response | 15 | 11 | 49 |
| 1. Antibody-dependent cell cytotoxicity | 21 | 14 | 74 |
| 1. Virus diseases | 22 | 38 | 169 |
| 1. Antigen presentation | 23 | 29 | 35 |
| 1. Immune complex clearance | 24 | 3 | 258 |
| 1. Cellular immunity | 25 | 24 | 39 |
| 1. Oral tolerance | 28 | 45 | 26 |
| 1. Complement activation | 29 | 8 | 30 |
| 1. Immune response | 30 | 27 | 31 |
| 1. Immunoglobulin secretion | 33 | 25 | 171 |
| 1. T-cell activation | 37 | 30 | 38 |
| 1. Lymphocyte proliferation | 39 | 65 | 56 |
| 1. Cytokine production | 40 | 35 | 6 |
| 1. Tolerance induction | 44 | 37 | 225 |
| 1. Insulin secretion | 51 | 57 | 47 |
| 1. Delayed hypersensitivity | 52 | 51 | 44 |
| 1. Regulation of immune response | 56 | 40 | 98 |
| 1. Adaptive immune response | 66 | 71 | 85 |
| 1. Virus replication | 70 | 84 | 72 |
| 1. Cell-mediated immune response | 89 | 79 | 134 |
| 1. Calcium signaling | 91 | 103 | 92 |
| 1. Cell movement | 96 | 129 | 110 |
| 1. Cytokine secretion | 104 | 96 | 22 |
| 1. Wound healing | 120 | 123 | 147 |
| 1. Tumor necrosis factor production | 147 | 135 | 4 |
| 1. Antigenic variation | 162 | 95 | 252 |
| 1. Transmembrane transport | 189 | 126 | 114 |
| 1. Ion transport | 194 | 128 | 127 |
| 1. Interleukin-10 production | 324 | 55 | 138 |
