# Supplementary figures and images for: Biosemantics guided gene expression profiling of Sjögren’s syndrome: a comparative analysis with systemic lupus erythematosus and rheumatoid arthritis
Source: Arthritis Res Ther. 2017 Aug 17;19:192. doi: 10.1186/s13075-017-1400-3 (PMC5561593; doi:10.1186/s13075-017-1400-3)

**S1**

## Analysis 1

(Fig. 1)

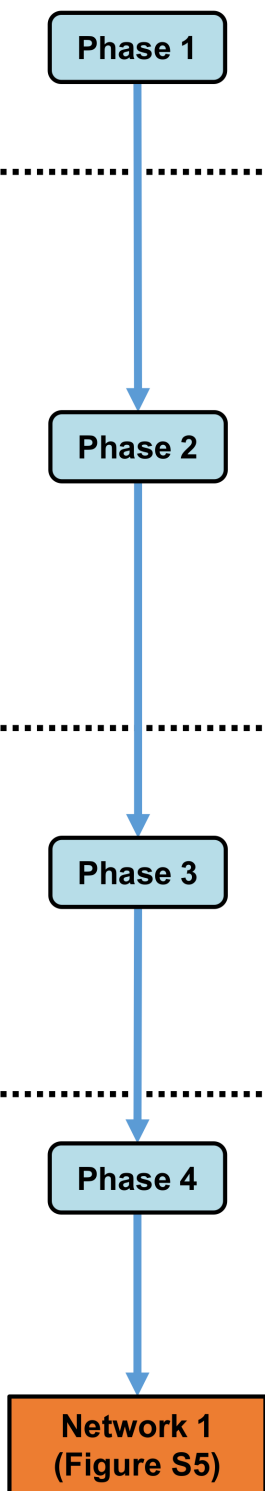

## Analysis 2

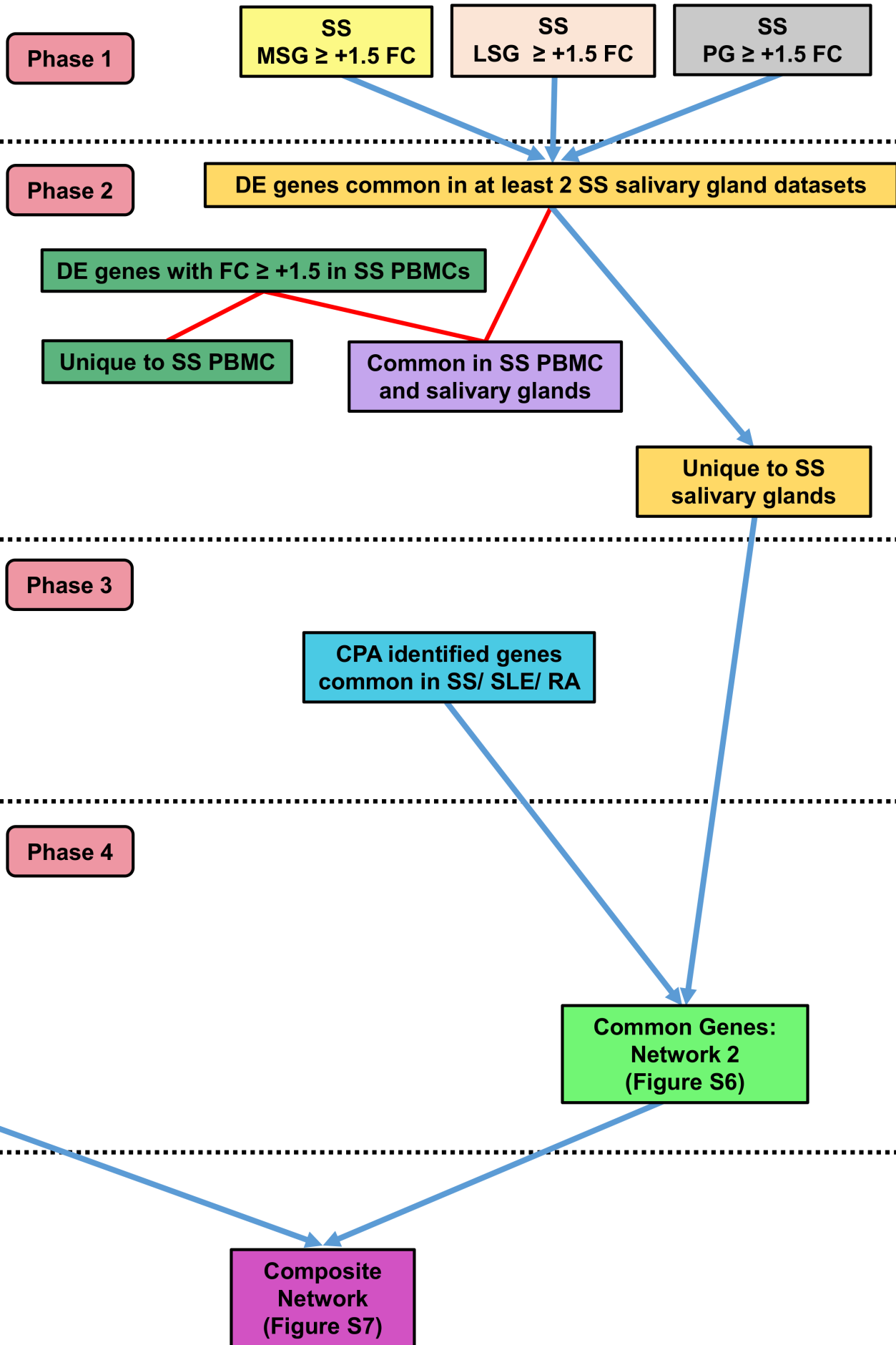

Supplement: Supplementary file 2 — Flowchart explaining analysis 2. (PDF 424 kb) [file 13075_2017_1400_MOESM2_ESM.pdf]

**S2**

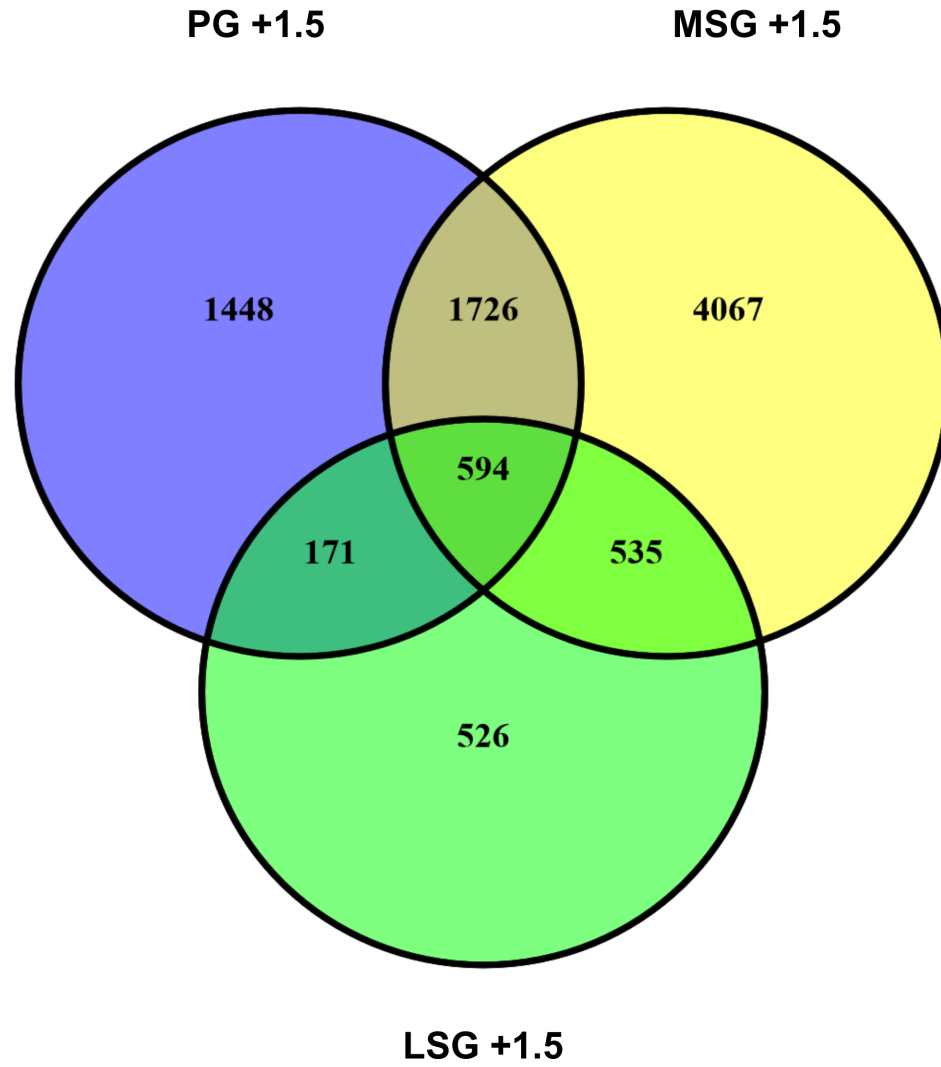

**Figure S2**

**S3**

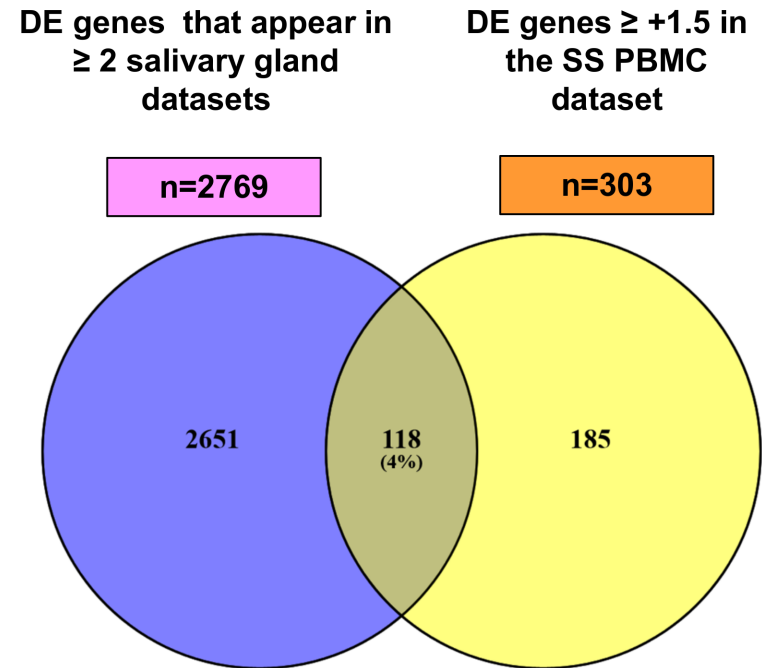

**Figure S3**

**S4**

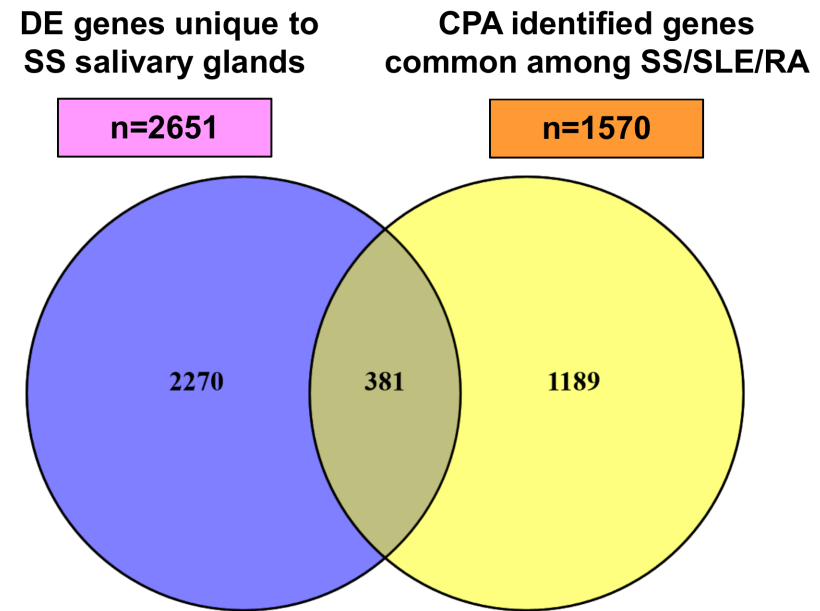

**Figure S4**

Supplement: Supplementary file 7 — Comparison of differentially expressed genes unique to salivary glands of SS female patients with CPA identified common genes from SS, SLE, and RA. (PDF 1003 kb) [file 13075_2017_1400_MOESM7_ESM.pdf]
