# Supplementary material for: Biosemantics guided gene expression profiling of Sjögren’s syndrome: a comparative analysis with systemic lupus erythematosus and rheumatoid arthritis
Source: Arthritis Res Ther. 2017 Aug 17;19:192. doi: 10.1186/s13075-017-1400-3 (PMC5561593; doi:10.1186/s13075-017-1400-3)
Supplement: Supplementary file 3 — Differential expression of SS and SLE CPA-identified common genes. (DOCX 79 kb) [file 13075_2017_1400_MOESM3_ESM.docx]

**Table S2.** SS and SLE CPA-identified common genes upregulated in PBMCs of SS patients and their differential expression in SS and SLE disease-site biological samples.

Table is showing the 41 SS PBMC genes (≥ +1.5 FC) in common with the 1849 SS and SLE common genes identified by CPA analysis and their respective FC values in three independent SS salivary gland and one SLE synovial biopsy datasets.

**Table S3.** SS and SLE CPA-identified common genes downregulated in PBMCs of SS patients and their differential expression in SS and SLE disease-site biological samples.

Table is showing the 13 SS PBMC genes (≤ -1.5 FC) in common with the 1849 SS and SLE common genes identified by CPA analysis and their respective FC values in three independent SS salivary gland and one SLE synovial biopsy datasets.

**Table S4.** SS and SLE CPA-identified common genes upregulated in PBMCs of SLE patients and their differential expression in SS and SLE disease-site biological samples.

Table is showing the 81 SLE PBMC genes (≥ +1.5 FC) in common the 1849 SS and SLE common genes identified by CPA analysis and their respective FC values in three independent SS salivary gland and one SLE synovial biopsy datasets.

**Table S5.** SS and SLE CPA-identified common genes downregulated in PBMCs of SLE patients and their differential expression in SS and SLE disease-site biological samples.

Table is showing the 52 SLE PBMC genes (≤ -1.5 FC) in common with the 1849 SS and SLE common genes identified by CPA analysis and their respective FC values in three independent SS salivary gland and one SLE synovial biopsy datasets.
