# Supplementary material for: Biosemantics guided gene expression profiling of Sjögren’s syndrome: a comparative analysis with systemic lupus erythematosus and rheumatoid arthritis
Source: Arthritis Res Ther. 2017 Aug 17;19:192. doi: 10.1186/s13075-017-1400-3 (PMC5561593; doi:10.1186/s13075-017-1400-3)
Supplement: Supplementary file 4 — Differential expression of SS and RA CPA-identified common genes. (DOCX 53 kb) [file 13075_2017_1400_MOESM4_ESM.docx]

**Table S6.** SS and RA CPA-identified common genes upregulated in PBMCs of SS patients and their differential expression in SS and RA disease-site biological samples.

Table is showing the 37 SS PBMC genes (≥ +1.5 FC) in common with the 1674 SS and RA common genes identified by CPA analysis and their respective FC values in three independent SS salivary gland and one RA synovial fluid datasets

**Table S7.** SS and RA CPA-identified common genes downregulated in PBMCs of SS patients and their differential expression in SS and RA disease-site biological samples.

Table is showing the 13 SS PBMC genes (≤ -1.5 FC) in common with the 1674 SS and RA common genes identified by CPA analysis and their respective FC values in three independent SS salivary gland and one RA synovial fluid datasets.

**Table S8.** SS and RA CPA-identified common genes upregulated in PBMCs of RA patients and their differential expression in SS and RA disease-site biological samples.

Table is showing the 27 RA PBMC genes (≥ +1.5 FC) in common with the 1674 SS and RA common genes identified by CPA analysis and their respective FC values in three independent SS salivary gland and one RA synovial fluid datasets.

**Table S9.** SS and RA CPA-identified common genes downregulated in PBMCs of RA patients and their differential expression in SS and RA disease-site biological samples.

Table is showing the 18 RA PBMC genes (≤ -1.5 FC) in common with the 1674 SS and RA common genes identified by CPA analysis and their respective FC values in three independent SS salivary gland and one RA synovial fluid datasets.
