# Supplementary material for: Biosemantics guided gene expression profiling of Sjögren’s syndrome: a comparative analysis with systemic lupus erythematosus and rheumatoid arthritis
Source: Arthritis Res Ther. 2017 Aug 17;19:192. doi: 10.1186/s13075-017-1400-3 (PMC5561593; doi:10.1186/s13075-017-1400-3)
Supplement: Supplementary file 5 — Differential expression of CPA-identified genes in salivary glands of patients with SS. (DOCX 40 kb) [file 13075_2017_1400_MOESM5_ESM.docx]

**Table S10.** Differential expression of CPA-identified genes in salivary glands of SS patients.

Summary of 76 genes differentially expressed (DE) in at least 2 out of 3 SS salivary gland (SG) microarray datasets displayed alphabetically. Genes with shaded grey color (n=22), are differentially expressed in at least 2 out of 3 SG datasets, but not in the third dataset in the opposite direction, and have not yet been described as being clearly associated with SS pathogenesis (*i.e.*, based on strong scientific evidence). Yellow background represents DE of that gene in a particular dataset (FC≤ -1.5 or FC≥ +1.5).
