# Supplementary material for: Biosemantics guided gene expression profiling of Sjögren’s syndrome: a comparative analysis with systemic lupus erythematosus and rheumatoid arthritis
Source: Arthritis Res Ther. 2017 Aug 17;19:192. doi: 10.1186/s13075-017-1400-3 (PMC5561593; doi:10.1186/s13075-017-1400-3)
Supplement: Supplementary file 6 — Differential expression of SLE and RA CPA-identified common genes. (DOCX 58 kb) [file 13075_2017_1400_MOESM6_ESM.docx]

**Table S11.** SLE and RA CPA-identified common genes upregulated in PBMCs of SS patients and their differential expression in SS, SLE and RA disease-site biological samples.

Table is showing the 66 SLE PBMC genes (≥ +1.5 FC) in common with the 1878 SLE and RA common genes identified by CPA analysis and their respective FC values in three independent SS salivary gland and one synovial fluid datasets.

**Table S12.** SLE and RA CPA-identified common genes upregulated in PBMCs of SS patients and their differential expression in SS, SLE and RA disease-site biological samples.

Table is showing the 24 SLE PBMC genes (≤ -1.5 FC) in common with the 1878 SLE and RA common genes identified by CPA analysis and their respective FC values in three independent SS salivary gland and one SLE synovial fluid datasets.

**Table S13.** SS and RA CPA-identified common genes upregulated in PBMCs of RA patients and their differential expression in SS and RA disease-site biological samples.

Table is showing the 9 RA PBMC genes (≥ +1.5 FC) in common with the 1878 SLE and RA common genes identified by CPA analysis and their respective FC values in three independent SS salivary gland and one RA synovial fibroblasts datasets.

**Table S14.** SS and RA CPA-identified common genes down-regulated in PBMCs of RA patients and their differential expression in SS and RA disease-site biological samples.

Table is showing the 5 RA PBMC genes (≤ -1.5 FC) in common with the 1878 SLE and RA common genes identified by CPA analysis and their respective FC values in three independent SS salivary gland and one RA synovial fibroblasts datasets.
