# Supplementary material for: Biosemantics guided gene expression profiling of Sjögren’s syndrome: a comparative analysis with systemic lupus erythematosus and rheumatoid arthritis
Source: Arthritis Res Ther. 2017 Aug 17;19:192. doi: 10.1186/s13075-017-1400-3 (PMC5561593; doi:10.1186/s13075-017-1400-3)
Supplement: Supplementary file 8 — Pathway analysis of genes common in the CPA analysis and upregulated in at least two out of three SS salivary gland datasets while not upregulated within PBMCs. (PDF 6363 kb) [file 13075_2017_1400_MOESM8_ESM.pdf]

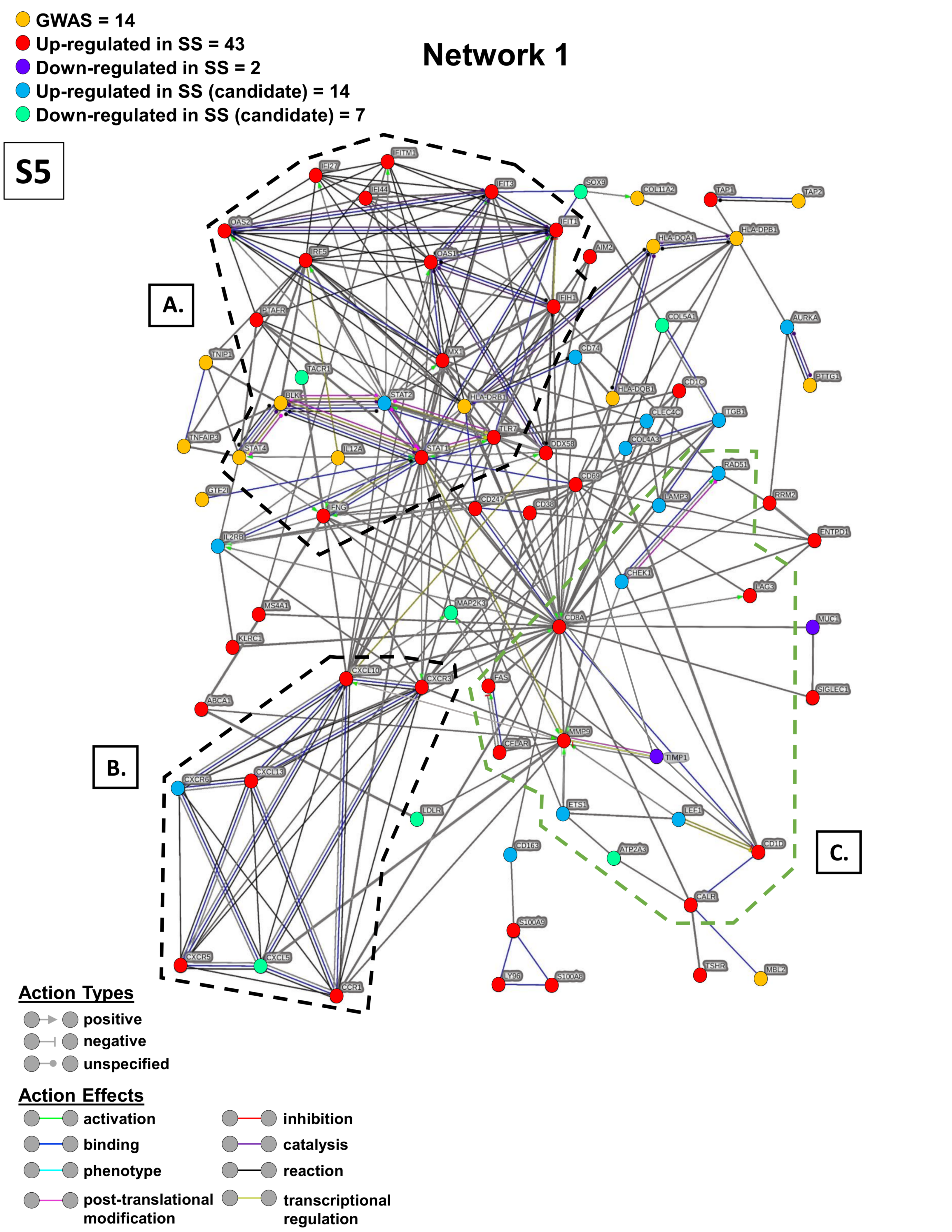
GWAS = 14
Up-regulated in SS = 43
Down-regulated in SS = 2
Up-regulated in SS (candidate) = 14
Down-regulated in SS (candidate) = 7
Network 1
S5
A.
B.
C.
IFITM1
IFI27
IFI44
IFIT3
SOX9
COL11A2
TAP1
TAP2
OAS2
IFIT1
AIM2
HLA-DQA1
HLA-DPB1
IRF5
OAS1
IFIH1
PTAFR
COL5A1
AURKA
CD74
MX1
TNIP1
TACR1
PTTG1
HLA-DQB1
CD1C
BLK
STAT2
HLA-DRB1
CLEC4C
ITGB1
TLR7
TNFAIP3
COL4A3
STAT4
IL12A
STAT1
DDX58
RAD51
CD69
GTF2I
IFNG
CD247
CD38
LAMP3
RRM2
ENTPD1
IL2RB
CHEK1
LAG3
MS4A1
MAP2K3
CD8A
MUC1
KLRC1
CXCL10
CXCR3
FAS
SIGLEC1
ABCA1
MMP9
CFLAR
TIMP1
CXCR6
CXCL13
LDLR
ETS1
LEF1
CD163
ATP2A3
CD1D
CALR
S100A9
CXCR5
CXCL5
CCR1
LY96
S100A8
TSHR
MBL2
Action Types
positive
negative
unspecified
Action Effects
activation
inhibition
binding
catalysis
phenotype
reaction
post-translational modification
transcriptional regulation
