# Supplementary material for: Biosemantics guided gene expression profiling of Sjögren’s syndrome: a comparative analysis with systemic lupus erythematosus and rheumatoid arthritis
Source: Arthritis Res Ther. 2017 Aug 17;19:192. doi: 10.1186/s13075-017-1400-3 (PMC5561593; doi:10.1186/s13075-017-1400-3)
Supplement: Supplementary file 9 — Pathway analysis of 76 selected differentially expressed genes with emphasis on three subnetworks. (PDF 9341 kb) [file 13075_2017_1400_MOESM9_ESM.pdf]

S6

Network 2

- Up-regulated in SS = 134
- Up-regulated in SS (candidate) = 7

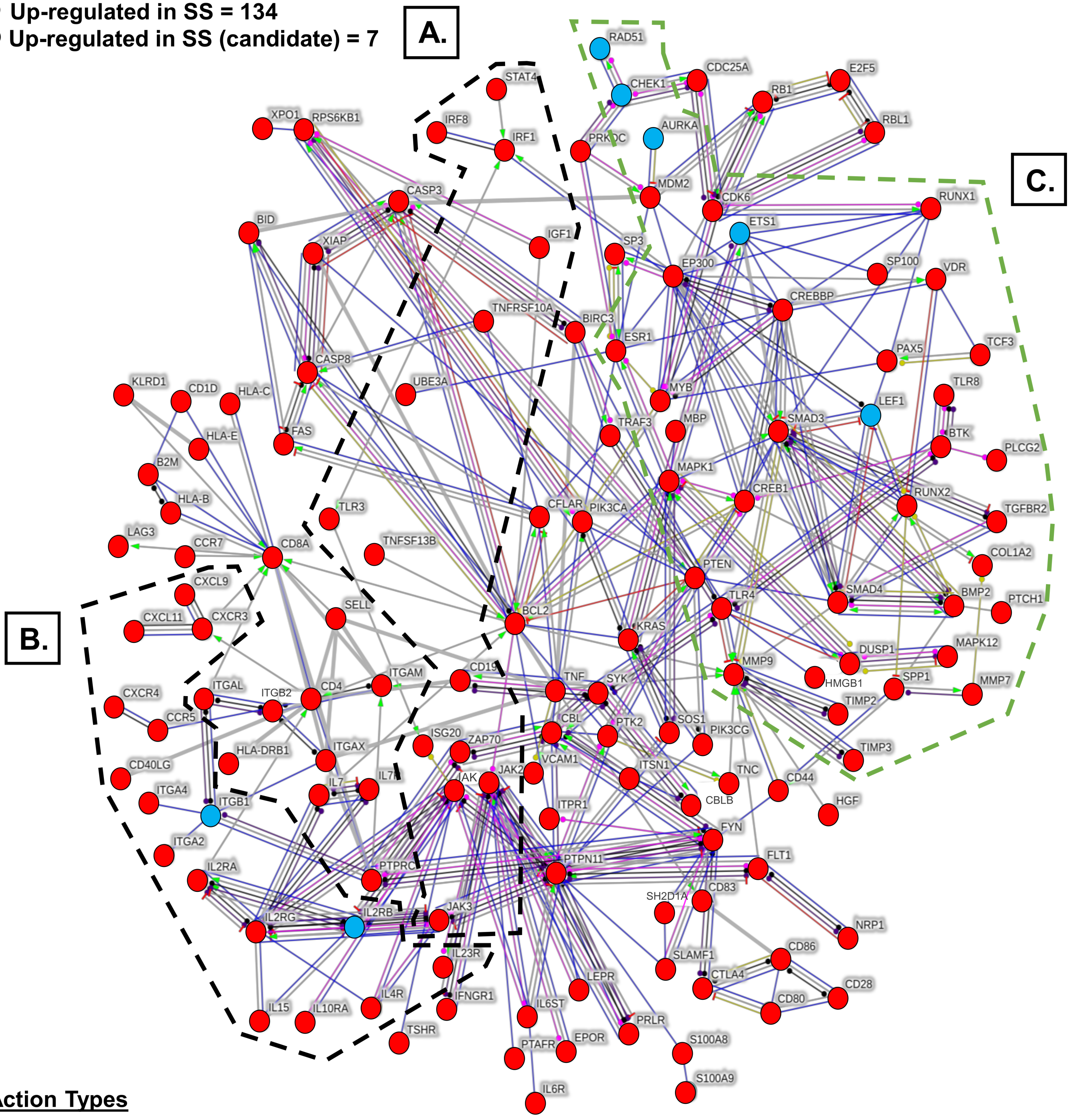

Action Types

- positive
- negative
- unspecified

Action Effects

- activation
- binding
- phenotype
- post-translational modification
- inhibition
- catalysis
- reaction
- transcriptional regulation
