# Supplementary material for: Biosemantics guided gene expression profiling of Sjögren’s syndrome: a comparative analysis with systemic lupus erythematosus and rheumatoid arthritis
Source: Arthritis Res Ther. 2017 Aug 17;19:192. doi: 10.1186/s13075-017-1400-3 (PMC5561593; doi:10.1186/s13075-017-1400-3)
Supplement: Supplementary file 10 — Composite network of subnetwork C from network 1 (Figure S4) and network 2 (Figure S6). (PDF 6025 kb) [file 13075_2017_1400_MOESM10_ESM.pdf]

S7

Composite Network

Network 1 = 10

Network 2 = 30

Common = 5

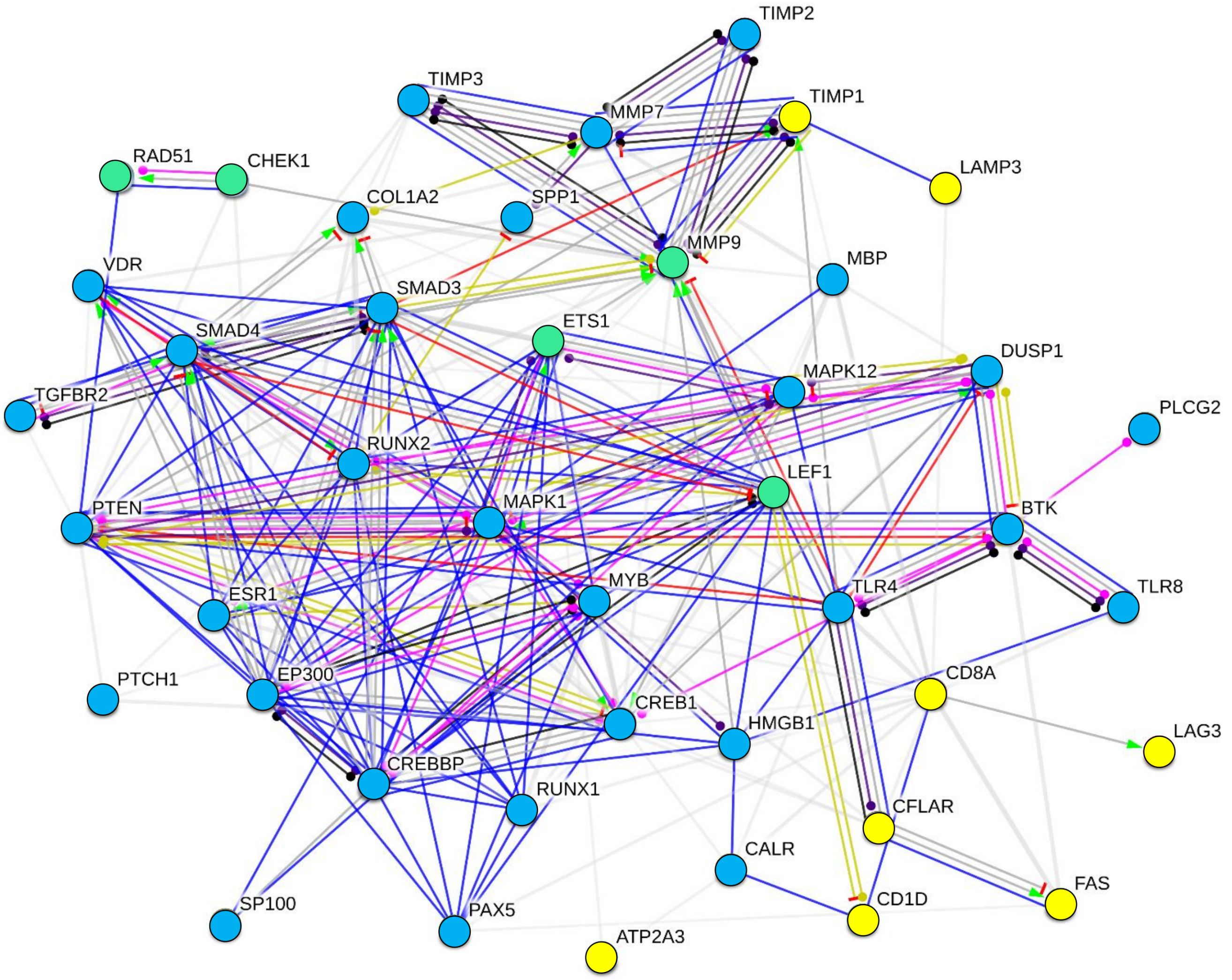

Network 1 (Subnetwork C. Figure S5) + **Network 2 (Subnetwork C. Figure S6)**

Action Types

- positive
- negative
- unspecified

Action Effects

- activation
- binding
- phenotype
- post-translational modification
- inhibition
- catalysis
- reaction
- transcriptional regulation
